# Ancillary Diagnostic Investigations in Malignant Pleural Mesothelioma

**DOI:** 10.3390/cancers13133291

**Published:** 2021-06-30

**Authors:** Alex Dipper, Nick Maskell, Anna Bibby

**Affiliations:** Academic Respiratory Unit, University of Bristol, Bristol BS105NB, UK; Nick.Maskell@bristol.ac.uk (N.M.); anna.bibby@bristol.ac.uk (A.B.)

**Keywords:** malignant pleural mesothelioma, pleural effusion, biomarkers

## Abstract

**Simple Summary:**

Malignant pleural mesothelioma (MPM) is a cancer affecting the covering of the lung (the pleura). This commonly causes a build-up of fluid around the lung, called a pleural effusion. Draining the pleural effusion can improve breathlessness and tests can be performed on the fluid. However, for most patients with MPM, a sample of tissue from the pleura, called a biopsy, is required in addition to make the diagnosis. Sometimes, due to medical conditions, frailty or personal preference, patients may not be able to have a biopsy. This review article discusses additional tests used in this situation to help doctors make a diagnosis of MPM. These techniques include tests on pleural fluid using “immunocytochemistry” methods, biomarkers and scans. Although, without a biopsy, no test in isolation can diagnose MPM, combining information from different types of tests and reviewing results among a specialist team can enable a consensus diagnosis.

**Abstract:**

For a number of patients presenting with an undiagnosed pleural effusion, frailty, medical co-morbidity or personal choice may preclude the use of pleural biopsy, the gold standard investigation for diagnosis of malignant pleural mesothelioma (MPM). In this review article, we outline the most recent evidence on ancillary diagnostic tests which may be used to support a diagnosis of MPM where histological samples cannot be obtained or where results are non-diagnostic. Immunocytochemical markers, molecular techniques, diagnostic biomarkers and imaging techniques are discussed. No adjunctive test has a sensitivity and specificity profile to support use in isolation; however, correlation of pleural fluid cytology with relevant radiology and supplementary biomarkers can enable an MDT-consensus clinico-radiological-cytological diagnosis to be made where further invasive tests are not possible or not appropriate. Diagnostic challenges surrounding non-epithelioid MPM are recognised, and there is a critical need for reliable and non-invasive investigative tools in this population.

## 1. Introduction

Arising predominantly from the pleural or peritoneal surface (less commonly the pericardium and tunica vaginalis), mesothelioma grows insidiously, often resulting in an advanced stage at clinical presentation. Whilst research into innovative treatment options is an active area of interest and brings new hope for patients, malignant pleural mesothelioma (MPM) remains relatively refractory to conventional therapies. Consequently, prognosis is poor, with a median survival of just 9.5 months and a 3-year survival rate of 12% [1,2].

An association with asbestos was first observed in 1960 in a case series of 33 patients with pleural mesothelioma from the Asbestos Hills in the Cape Province of South Africa [3]. Today, 85% of all mesotheliomas in males are attributable to occupational asbestos exposure, with para-occupational exposure being a recognised cause in women [4]. Despite a ban on asbestos products in 52 countries by 2010 [5], the long latency period from exposure to disease (typically 30–40 years) and continued unregulated use in countries such as India, Brazil and Russia means that MPM continues to represent a significant global health concern, with an estimated burden of 38,400 cases per year worldwide [6].

Other aetiological mechanisms include genetic predisposition, with inherited germline mutations of the BRCA 1-associated protein (BAP1) gene (a tumour suppressor gene involved in modulation of transcription and DNA repair) identified amongst families with high incidence of mesothelioma in 2011 [7]. Exposure to other elongated mineral particles (including environmental exposure to erionite and fluoro-edenite in Turkey, USA and Mexico) and ionising radiation are also implicated [8]. Pathogenic mechanisms of carcinogenesis following asbestos fibre inhalation highlight a cycle of genetic and cellular damage with chronic inflammation [2,4,9,10,11].

Four main histological subtypes of MPM are described; epithelioid, sarcomatoid, biphasic and desmoplastic, with epithelioid associated with the most favourable prognosis (median survival of 13 months) and sarcomatoid the least (median survival 4 months) [12]. With no established role for surgical resection outside of clinical trials [1], histological diagnosis of MPM typically relies on biopsy samples. Thoracoscopic pleural biopsy is recommended as the gold standard for investigating an undiagnosed pleural effusion where the differential includes MPM, with diagnostic yields of 95% and higher [13]. Alternatively, where contrast-enhanced thoracic computed tomography (CT) demonstrates focal areas of abnormal pleura, image-guided needle biopsy may be employed to obtain tissue [8,14,15].

There is a cohort of patients for whom frailty, medical co-morbidity or personal choice preclude the use of invasive pleural biopsy. The 2020 UK National Mesothelioma Report showed the median age of patients diagnosed with pleural mesothelioma was 76 years and that over 20% of patients had stage IV disease at diagnosis [16]. Furthermore, a multinational population-based evaluation of 9014 patients demonstrated that more than half of those diagnosed with mesothelioma were aged 70 years or older [17]. Given demographic trends, the proportion of elderly patients will continue to rise over coming decades, with increasing comorbidity further complicated by advanced stage at disease presentation. Diagnostic approaches that are tolerable to and appropriate for patients of higher age or with significant comorbidity are increasingly necessary. Additionally, a proportion of patients who are considered suitable to undergo pleural biopsy at initial assessment go on to have a protracted diagnostic pathway, with repeated procedures yielding equivocal or non-diagnostic results.

Although international guidelines do not advocate cytology-based diagnoses of MPM in patients who are fit for further diagnostic tests [1], the importance of obtaining a diagnosis for frail patients who are unable to undergo invasive procedures to obtain a biopsy is no less significant. Confirmation of a diagnosis is important for future planning and to enable patients to access financial compensation. In some regions, a multi-disciplinary team (MDT) diagnosis based on cytological, radiological and clinical information is sufficient to avoid requirements for a post-mortem examination after death [18].

In this article, we will explore and outline the most up-to-date evidence on ancillary diagnostic tests currently available in clinical practice. We will focus on techniques which may be used to support a diagnosis of MPM from cytological specimens and other less invasive modalities, where histological samples cannot be obtained or where results may be non-diagnostic.

## 2. Pleural Fluid (PF) Cytology

Diagnostic thoracentesis is the primary means of obtaining PF for evaluation and is an essential step in the initial investigation of a unilateral pleural effusion [19]. Diagnostic cytology on PF can spare the patient more invasive investigations to obtain a tissue biopsy, reducing the risk of procedural complication with both cost and time saving in addition. However, the diagnostic yield of MPM from conventional PF cytology alone is highly variable, with sensitivity ranging from 16% to 73% [1]. In one study of 921 patients with an undiagnosed unilateral pleural effusion, fluid cytology was diagnostic in only 9 of 148 (6%) participants with MPM [20].

Several factors contribute to the wide range of sensitivities quoted. Whilst epithelioid cancers can shed malignant cells into pleural effusion fluid, this is rare in sarcomatoid subtypes. Cytological diagnosis is usually limited, therefore, to the epithelioid subtype. Heavy bloodstaining or rich inflammatory cell infiltrate may additionally reduce cellular yield in effusion specimens. Concentration techniques such as cell block and cytospin preparations can overcome these problems and enhance detection of malignant cells. Cell blocks can also provide a substrate on which adjunctive tests, including immunocytochemical and molecular techniques, can be applied. [8,21,22]

Cytologist experience is another important consideration, with cytopathology being a recognised subspecialty in its own right. For example, morphological appearances of benign reactive mesothelial cells can overlap with malignant cells, complicating diagnosis and demanding meticulous assessment. The volume of PF submitted for analysis may be an additional limitation [1,23], with the British Thoracic Society recommending that 20–40 mL should be sent for evaluation [19].

An important limitation on cytology-based diagnosis is the inability to determine tumour invasion into the lung or chest wall on the basis of PF cytology alone [21,24]. Cytological yield in epithelioid mesothelioma is, however, higher in the presence of visceral pleural invasion. In one study of 75 patients with epithelioid MPM, 37/45 (82%) with positive PF cytology at initial thoracentesis had evidence of visceral pleural invasion at local anaesthetic thoracoscopy (defined as masses, nodules, thickening or mixed appearance) compared with 9/30 (30%) patients having negative cytology, giving an odds ratio for an association between visceral pleural invasion and cytological positivity of 11.87 (95% confidence interval (CI): not stated; *p* < 0.001) [25].

## 3. PF Immunohistochemistry (IHC) and Molecular Techniques

Initial cytomorphology may be sufficient to confirm the presence of malignant cells in PF after routine staining and, in some cases, may confirm MPM. However, more often, ancillary techniques are required to discriminate benign from malignant mesothelial populations and to differentiate MPM from carcinoma or neoplasms of other origins (for example, melanoma). Recent advances in immunocytochemical and molecular testing have facilitated these diagnostic steps [22,26].

### 3.1. Discriminating Benign from Malignant Mesothelial Populations

Reactive mesothelial proliferation is a common mimic of MPM (and metastatic carcinoma) and has numerous causes, including infection, pulmonary infarction, trauma, autoimmune disease and drug reactions [27]. Cytomorphological features overlap with MPM and include high cellularity, numerous mitotic figures and cytologic atypia. The inability to evaluate tissue invasion in cytology-based specimens means that reactive mesothelial proliferation is more frequently documented in cytologic specimens than in tissue biopsies [24].

Certain immunocytochemical stains are more likely to be positive in benign mesothelial cell proliferation and other stains in malignant mesothelial proliferation. However, most IHC staining patterns do not reliably differentiate malignant from benign mesothelial proliferation. Desmin, reported previously to favour benign reactive mesothelium, shows positivity in up to 56% of mesotheliomas [28]. Similarly, whilst epithelial membrane antigen (EMA), p53 and insulin-like growth-factor 2 messenger ribonucleic acid (RNA)-binding protein 3 (IMP-3) may support a diagnosis of malignancy, benign reactions can also stain positively for these markers [29]. Whilst positive staining with glucose transporter 1 (GLUT1) may have a higher specificity for malignant cell populations in pleural biopsy specimens [1], cytological studies demonstrate lower specificity, with 9/50 patients with benign reactive mesothelial proliferations demonstrating positive polyclonal GLUT-1 staining in one study [30] and 14/38 participants with benign effusions staining positive in another [31].

Detection of specific mesothelioma-associated genetic mutations can help confirm the presence of malignant cells. Loss of BAP1 can be demonstrated on IHC staining and is highly specific for malignancy, whilst fluorescent in situ hybridisation (FISH) can detect deletion of the *CDKN2A/P16* gene, commonly seen in MPM.

#### 3.1.1. BAP1 Loss

BAP1 is a nuclear ubiquitin hydrolase, which functions as a tumour suppressor, and is encoded by the BAP1 gene. It controls DNA repair, expression of genes related to cell cycle and cell proliferation. It can also induce cell death. Cells with reduced or absent BAP1 are unable to repair damaged DNA and cannot execute apoptosis. BAP1-mutant cells are therefore prone to malignant transformation [10].

Somatic mutation of the BAP1 gene in mesothelioma was first described in 2011, with mutations occurring in approximately 70% of epithelioid mesotheliomas [10]. Germline BAP1 mutation is less common, occurring in approximately 1–2% of MPM, usually in the context of the autosomal dominant BAP1 cancer predisposition syndrome [29,32]. Germline BAP1 loss is associated with earlier onset MPM tumours, as well as other BAP1-related malignancies such as uveal melanoma.

BAP1 loss (defined as absence of nuclear staining when a positive internal control is present on a slide) may occur by mutation, biallelic deletion or deletion/insertion [8] and is most reliably detected by IHC [32]. Cells expressing at least one wild-type copy of BAP1 retain IHC staining. Notably, even in tumours arising from germline BAP1 mutation, non-tumour cells express a single wild-type copy and hence produce a positive IHC response. To show loss of BAP1 immunoreactivity, both copies must be mutated, either by a combination of germline and somatic mutation events, as in BAP1 cancer syndrome, or by two somatic events in sporadic cancers [29].

Loss of BAP1 expression has been repeatedly validated in differentiating MPM from benign mesothelial populations and is now in routine use in many pathology laboratories. A recent meta-analysis identified 12 studies of 1824 patients (1016 with MPM), published between 2015 and 2017. The overall pooled sensitivity of BAP1 loss for malignant mesothelioma was 0.56 (95% CI: 0.50–0.62) and specificity 1.00 (95% CI: 0.95–1.00). The area under curve (AUC) was 0.72, indicating moderate diagnostic accuracy. Notably, all studies were of retrospective design, and only four included more than 100 participants. Heterogeneity was evident, with potential explanations including different cut-off values for BAP1 loss, inclusion of participants with pleural and peritoneal mesothelioma and variation in diagnostic accuracy across mesothelioma histological subtypes. For example, the sensitivity ranged from 0.07 (95% CI: 0.00–0.72) in sarcomatoid MPM to 0.74 (95% CI: 0.66–0.80) in epithelioid [33]. Offering additional explanation for this low diagnostic sensitivity, 30–40% of mesotheliomas have been shown to carry a wild-type BAP1 and therefore stain positively in a similar manner to benign lesions [10].

In a subgroup meta-analysis comparing the diagnostic performance of BAP1 loss in histology and cytology specimens, near identical sensitivity and specificity was observed. However, data from the 5 studies evaluating cytology specimens demonstrated reduced diagnostic accuracy with an AUC of 0.69 [33]. Studies of BAP1 loss in cytology specimens have, to date, been hindered by retrospective design, small sample size and the use of cytology specimens in subgroup analyses. Well-designed research is required to accurately determine the diagnostic potential of BAP1 loss in cytology specimens in order to improve current diagnostic pathways and potentially avoid the need for additional invasive procedures.

As a stand-alone test, BAP1 loss has moderate diagnostic sensitivity with excellent specificity for MPM. BAP1 loss is therefore reliable as a “rule in” for mesothelioma, but pleural malignancy cannot be excluded in its absence. Notably, BAP1 loss is uncommon in sarcomatoid and desmoplastic mesothelioma and is demonstrated in other malignancies including melanoma and renal cell carcinoma [34]. Superior diagnostic accuracy may be achieved in combination with other adjunctive tests.

#### 3.1.2. p16 Fluorescence In Situ Hybridization (FISH)

Homozygous deletion of the 9p21 locus is one of the most common genetic alterations in MPM. Its loss affects a cluster of genes, including p16 (also known as cyclin-dependent kinase inhibitor (CDKN)-2A), CDKN2B and methylthioadenosine phosphorylase (MTAP). p16/CDKN2A is a tumour suppressor gene that is present in all healthy cells. Its normal function results in the cessation of the cell cycle; hence, inactivation results in uncontrolled cell proliferation and tumour development.

Homozygous deletion of P16 can be detected using FISH in both cytological and histological specimens [35]; however, the diagnostic sensitivity for MPM is relatively low at 0.53 (95% CI: 0.35–0.70), despite gene profiling studies demonstrating p16/CDKN2A loss in up to 80% of MPM tumours. In part, the low sensitivity reflects variation in p16 deletion across the different MPM subtypes (90–100% loss in sarcomatoid variant compared with a 70% loss in epithelioid and biphasic), although other alterations that affect the 9p21 locus and cannot be detected by FISH also contribute [1,8,21,23,24,29].

An alternative approach, where histological specimens are available, is the application of IHC staining to determine p16 protein expression in cells, which could represent a more accessible ancillary test to laboratories where FISH cannot be performed [36]. However, the sensitivity to discriminate MPM from reactive mesothelial hyperplasia using p16 IHC in combination with BAP1 loss was 10% lower than those of more traditional FISH techniques in one study [35]. IHC techniques may be employed, in addition, to detect MTAP loss, distinguishing malignant from benign proliferations with a specificity of 100% and a sensitivity of 43% (increased to 79.5% when used in combination with BAP1 IHC) in cell block specimens from pleural effusions [37,38]. IHC for MTAP can also discriminate sarcomatoid MPM from fibrous pleuritis. A more recent multicentre evaluation of MTAP loss by IHC demonstrated a 78% sensitivity and a 96% specificity for CDKN2A homozygous deletion, suggesting it to be a reliable surrogate for CDKN2A FISH [39]; however, the use of MTAP is not yet recommended by international guidelines [1,8,15].

**Figure 1:**
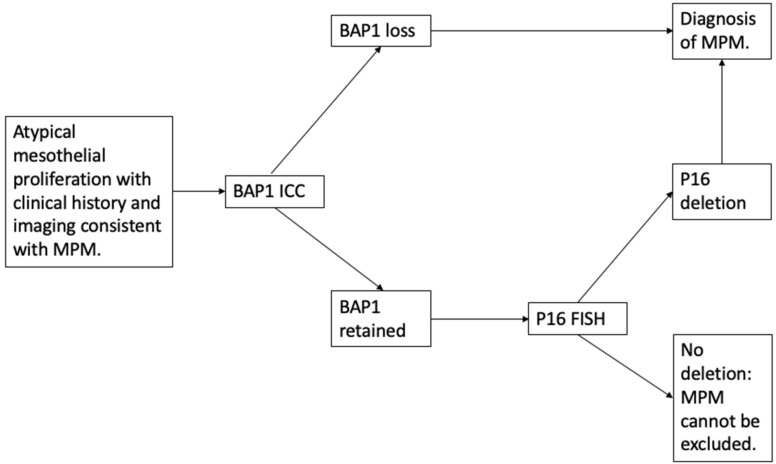
A suggested diagnostic approach where distinction of malignant from benign mesothelial proliferation is unclear on initial fluid cytology. BAP1 loss and p16 deletion support the diagnosis of MPM. MPM, malignant pleural mesothelioma; ICC, immunocytochemistry; FISH, fluorescence in-situ hybridization; BAP1, BRCA 1-associated protein.

Overall, when used in isolation, both FISH and IHC techniques for p16 deletion are limited by low sensitivity. Consequently, whilst p16 deletion can confirm a suspected diagnosis of malignancy, failure to detect its loss does not exclude a diagnosis of MPM. However, combining testing for p16 loss with IHC for BAP1 loss has been shown to increase diagnostic sensitivity (combined sensitivity 0.76 (95% CI: 0.62–0.88)) [40]. Therefore, if BAP1 is intact or a sarcomatoid mesothelioma is suspected, additional testing with p16 FISH may strengthen diagnostic certainty [21] and help to discriminate benign from malignant mesothelial cell populations (Figure 1).

### 3.2. Distinguishing Mesothelioma from Carcinoma

Distinguishing mesothelioma from other causes of malignant pleural effusion is critical in guiding therapeutic strategies and prognosis. Malignancies commonly metastasising to the pleura include lung cancers, breast and gastrointestinal carcinomas. Distinction between epithelioid MPM and carcinomas may be made on morphology and simple histochemical staining alone. As no one marker exhibits a 100% specificity, guidelines recommend a combination of at least two positive mesothelial markers (calretinin, cytokeratin 5/6, Wilms tumour 1 and D2-40) and at least two negative adenocarcinoma IHC markers (thyroid transcription factor 1 (TTF1), carcinoembryonic antigen (CEA) and Ber-EP4) (see Table 1) [1]. Positive markers of other tumour types should be used for differential diagnoses of metastatic carcinomas from other sources, such as hormone receptors in breast and ovarian cancer and PAX8 in renal cell carcinoma [1,15,24].

BAP1 loss may play a role in differentiating mesothelioma from carcinoma, with loss in 46/53 (87%) pleural and peritoneal mesotheliomas compared with 4/204 (2%) (*p*: <0.001) carcinomas in one study [41]. Further evaluation of the role of BAP1 loss in this context is required, however, before universal adoption is recommended.

### 3.3. Distinguishing Mesothelioma from Other Malignant Cell Neoplasms

Malignant pleural effusion may be the first presentation of an unknown primary cancer. In this setting, appropriate immunocytochemical panels often enable a precise diagnosis, starting with CK7 and CK20 staining [42]. Other differential diagnoses of MPM depend on histologic category, with epithelioid MPM requiring distinction from carcinomas, sarcomatoid MPM from sarcomas and other spindle cell neoplasms, mixed MPM from other mixed or biphasic tumours such as synovial sarcoma and desmoplastic MPM from fibrous pleuritis. Immunostain selection in this setting would depend on basic morphology [24].

Affirmative markers used in the evaluation of epithelioid MPM are of limited utility in sarcomatoid tumours. More usefully, cytokeratin markers, such as CAM5.2, are important in differentiating sarcomatoid MPM (positive staining) from sarcoma, which is usually keratin-negative [43]. D2-40 (podoplanin) can be used to differentiate sarcomatoid MPM from pulmonary sarcomatoid carcinoma (which also stains positively for TTF1, napsin and p40/p63). Synovial sarcoma can be confirmed by molecular testing for the X;18 translocation [24].

Immunocytochemical markers are summarised in Table 1. 

## 4. Diagnostic Biomarkers

Biomarkers present an attractive solution to diagnostic challenges posed by MPM, and consequently, a large number of studies have evaluated potential targets in serum, plasma, PF and exhaled breath. An ideal marker should be obtainable by minimally invasive means and be sufficiently sensitive to detect most cases of MPM, whilst also being highly specific, to avoid false positive results and discriminate individuals with MPM from other pathologies. Protein biomarkers of interest include mesothelin, osteopontin and fibulin-3 [45].

### 4.1. Mesothelin

Identified in the early 1990s as a surface antigen on ovarian cancer cells, mesothelin is a glycoprotein thought to play a role in cell adhesion and signalling. The mesothelin gene, MSLN, encodes a precursor protein from which membrane-bound mesothelin and a soluble protein megakaryocyte potentiating factor (MPF) are formed. These are commonly referred to as “soluble mesothelin-related peptides” (SMRPs). In normal tissue, mesothelin is only found on mesothelial cells; hence, serum levels of SMRP are low. However, increased concentrations of SMRPs are found in serum samples of patients with ovarian and pancreatic cancers, in addition to mesothelioma. In 2003, Robinson et al. demonstrated that patients with MPM had significantly higher concentrations of serum SMRP than asbestos-exposed healthy controls, non-asbestos-exposed healthy controls and patients with non-mesothelioma malignant or inflammatory pleural disease. They reported a sensitivity of 84% (95% CI: 73–93) and a specificity of 100% (95% CI: 91–100) for MPM. SMRP concentrations were higher in patients with epithelioid tumours and in those with a large tumour bulk (maximum tumour width: >3 cm) [11,46,47]. In contrast, SMRP was less likely to be raised in people with sarcomatoid and biphasic disease; however, small study numbers and non-disclosure of histologic subtype in some studies mean that accurate sensitivity and specificity estimates are difficult to derive for these tumour subtypes [11].

Serum mesothelin has become the most widely studied diagnostic biomarker in MPM, with a meta-analysis in 2014 identifying 28 relevant publications, involving 7550 patients [48]. Pooled sensitivity and specificity estimates were found to be 0.61 and 0.87, respectively, lower than indicated in previous studies. This is mostly accounted for by heterogeneity across the included studies, although publication bias may also play a role. Heterogeneity arose from the use of various ELISA assays, different cut-off values and differences in participant characteristics (i.e., mesothelioma subtypes and choice of control groups). The negative likelihood ratio (NLR) value was 0.43, meaning if participants were serum-SMRP-negative, the probability of having MPM was still moderate at 43%. The authors reported that low sensitivity limited the added value of SMRPs but a positive result may be helpful in confirming MPM, with a positive likelihood ratio of 5.71 [48].

PF mesothelin has been studied as an alternative biomarker, as mesothelin is shed from mesothelioma tumour cells directly into pleural effusion fluid. In 2005, Pass et al. identified that SMRP levels were significantly higher in PF samples from 45 patients with MPM compared to 30 healthy controls [49]. In the first study to assess the clinical utility of PF SMRP, Davies et al. demonstrated levels were 10.9 times greater in patients with MPM compared to benign pleural disease and were highly reproducible [50]. They concluded that the measurement of PF mesothelin contributed valuable additional information to PF cytology alone, especially where initial cytology results were inconclusive. In a meta-analysis by Cui et al., pooled estimates of sensitivity were higher for PF SMRP than serum samples (0.79 compared to 0.61) with PF SMRP specificity remaining robust at 0.85 [48].

Although considered as the current “gold standard” biomarker for MPM in some international guidelines [15], neither serum nor pleural fluid mesothelin is recommended as diagnostic tests in isolation. With low sensitivity, a negative result adds little value and is a frequent finding in non-epithelioid disease. In contrast, a positive result increases the likelihood of mesothelioma; however, false positives are possible in benign inflammatory conditions such as benign asbestos pleural effusion (BAPE) or in the presence of impaired renal function [51]. Consequently, mesothelin testing should be considered as an adjunct in patients with suspicious or inconclusive cytology, who are unsuitable for or decline invasive diagnostic tests with a high pre-test probability of MPM [1,4,8]. Further research into the utility of biomarkers in MPM diagnosis and better understanding of markers of non-epithelioid disease may help to elucidate the role of this test in the diagnostic pathway.

### 4.2. Other Diagnostic Biomarkers

Osteopontin, a protein mediator of cell matrix interaction, cell signalling and tumour development, has been viewed as a promising biomarker for MPM, but results have been inconsistent. In a meta-analysis of six studies, the overall diagnostic sensitivity and specificity were 0.65 (95% CI: 0.6–7.0) and 0.81 (95% CI: 0.78–0.85), respectively. Notably, the majority of included studies evaluated serum and/or plasma osteopontin from frozen samples with uncertainty regarding the long-term stability of osteopontin in frozen specimens. Degradation of osteopontin during the freezing and defrosting process may explain the low detection rates of this protein in retrospective studies [52]. Similar to mesothelin, the clinical utility of osteopontin is limited by low sensitivity, and further understanding of its added diagnostic value in comparison to other biomarkers is required.

Fibulin 3, an extracellular matrix glycoprotein mediator of cell-to-cell and cell-to-matrix communication, is detectable in blood and PF with a small number of studies reporting varied outcomes on its potential as a biomarker for MPM. Initially promising, with a 97% sensitivity and a 95% specificity to determine MPM from other causes of pleural effusion in one study [53], subsequent analyses have suggested a sensitivity as low as 22% [54]. A questionable diagnostic value was highlighted by one study, with no difference in fibulin 3 levels in pleural effusion samples of patients with MPM and controls. Whilst plasma levels were higher in patients with MPM compared to in controls in a population in Sydney, this was not replicated in a cohort of patients studied in Vienna and the diagnostic accuracy was low (receiver operating curve analyses overall accuracies of 63.2% and 56.2% for correct diagnostic characterisation of MPM in the Sydney and Vienna cohort, respectively). The authors did, however, observe that low pleural effusion fibulin 3 levels were significantly associated with better survival [55]. A meta-analysis of 8 studies demonstrated a pooled diagnostic sensitivity of blood fibulin 3 of 0.87 (95% CI: 0.58–0.97) and a specificity of 0.89 (95% CI: 0.77–0.95) [56]. A subsequent meta-analysis of 7 studies demonstrated a lower overall sensitivity from pooled studies of blood and pleural effusion samples of 0.62 (95% CI: 0.45–0.77) and a specificity of 0.82 (95% CI: 0.73–0.89) [57]. Ultimately the value of fibulin 3 in diagnosing MPM remains unclear, with prospective validation studies ongoing [58].

## 5. Imaging Techniques

CT with contrast enhancement is the primary imaging modality used for diagnosis and staging of pleural malignancy and can identify the primary tumour, intrathoracic lymphadenopathy and extrathoracic spread [59]. Positive features of malignant pleural disease include circumferential pleural thickening, nodular pleural thickening, parietal pleural thickening of greater than 1 cm and mediastinal pleural involvement [60]. The diagnostic accuracies of CT for detection of pleural malignancy are 68–97% with specificities of 78–89% [1]. CT scanning is widely available and has high clinical utility. However, it has limited soft tissue differentiation, and early malignant disease with minor pleural thickening can be missed. Additionally, subtle invasion of certain structures may be challenging to identify, which has implications for the accuracy of staging. Timing of contrast and reporting of images by non-thoracic radiologists add further variability. Subsequently, 35–46% of patients with pleural malignancy will have a “benign” CT report in routine practice [61].

Differentiating mesothelioma from metastatic pleural malignancy can also be challenging. Parenchymal lung tumours with mediastinal or hilar lymphadenopathy may indicate metastatic pleural disease, whereas the presence of pleural plaques, involvement of the interlobar fissure and absence of lung parenchymal masses favour MPM [1]. It may be particularly difficult to differentiate MPM from pleural metastatic disease, if the tumour presents as a localised pleural or subpleural nodule, a localised anterior mediastinal mass or involves the diaphragmatic pleura with liver invasion, especially in the absence of a pleural effusion [43].

Alternative imaging modalities have been proposed for use in MPM. Positron emission technology (PET)-CT combines high-resolution CT scanning with an injection of a metabolic tracer which accumulates at areas of metabolic activity. Uptake is assessed at regions of interest and reported as standard uptake values (SUV), with a threshold value of 2.0 reliably differentiating between benign and malignant disease [4]. A meta-analysis of 11 PET-CT studies reported a pooled sensitivity of 95% (95% CI: 92–97%) and a specificity of 82% (95% CI: 76–88%) for differentiating malignant from benign disease [62]. False positive results are common, however, particularly in the context of prior talc pleurodesis, active pleural infection, or indolent inflammation such as tuberculous pleuritis. PET-CT cannot distinguish MPM from metastatic pleural disease and, due to poor spatial resolution, has low sensitivity (78%) for extrapleural invasion [61]. Whilst lacking specificity to diagnose MPM routinely, PET-CT may provide functional information on pleural lesions, although it does not appear to be helpful in guiding choice of site for biopsy [63]. It is currently recommended only for staging patients in whom the presence of distant metastatic disease would alter treatment approach [1,8,15].

Magnetic resonance imaging (MRI) offers higher soft tissue contrast than CT, resulting in an increased sensitivity for chest wall and diaphragm invasion, higher contrast with adjacent effusion and higher inter-observer agreement [64]. The contrast enhanced perfusion augments sensitivity in detection of pleural malignancy, even where pleural thickening is minimal [64]. In addition to differentiating malignant from benign pleural disease, diffusion-weighted MRI (DWI-MRI) has distinguished between epithelioid and sarcomatoid MPM with a sensitivity of 60% and a specificity of 94% [1]. At present, the added value of MRI in equivocal or atypical CT scans is unclear, with prospective evaluation required, but, where available, MRI may be considered in difficult diagnostic cases to better delineate invasive disease [1,8,15].

## 6. Future Directions

The search for novel diagnostic biomarkers is expanding and encompasses multiple branches of medical science. Proteomic analysis has identified new panels of candidate biomarkers [65] with prospective multicentre evaluation of a novel assay ongoing [58]. Gene-expression-based classification has outperformed BAP1 and p16 FISH [40]. Deeper understanding of the genomic and epigenomic factors relevant to MPM may herald new diagnostic techniques that better distinguish MPM from other tumours [66,67,68]. Circulating plasma micro-RNA [69] and metabolomic profiling [70,71] of PF are other experimental areas of interest.

Whilst these studies may yield new markers which negate the requirement for invasive tissue sampling, all are limited currently to the research setting and are not yet available in clinical practice.

As the range of therapeutic options for MPM expands, the importance of genetic and molecular phenotyping of tumours to enable targeted treatment will increase. Currently, no marker is able to provide this level of personalised tumour phenotyping, so tissue biopsies are likely to remain the diagnostic gold standard for the foreseeable future.

To obtain tissue in patients fit to undergo invasive procedures, a “direct-to-LAT” approach (pathway stratification where selected patients proceed directly to local anaesthetic thoracoscopy (LAT) to obtain pleural biopsies) may be employed in patients where the pre-test probability of MPM is high and the anticipated yield from PF cytology is low [72]. However, a streamlined diagnostic approach is required for more frail patients and those who choose not to undergo pleural biopsy. Research to determine the combined value of the investigations discussed in this article is essential to formalise integrated non-invasive pathways for the diagnosis of MPM.

## 7. Conclusions

For patients in whom malignant pleural mesothelioma is suspected, tissue diagnosis remains the gold standard and is the only method that can confirm the presence of invasive disease. However, for those unable or unwilling to undergo tissue sampling, the low sensitivity of pleural effusion cytology can be augmented by incorporating ancillary techniques such as immunocytochemical markers to increase reliability [8]. No adjunctive test has a sensitivity and specificity profile to support use in isolation, but findings such as BAP1 loss can provide additional support for a suspected diagnosis if the pre-test probability is high. Where diagnoses remain challenging, even despite use of ancillary techniques, expert radiological review of disease distribution on imaging and occupational history of asbestos exposure are important considerations. Correlation of PF cytology with relevant radiology and supplementary biomarkers can enable an MDT-consensus clinico-radiological-cytological diagnosis to be made, where further invasive tests are not possible or not appropriate [18]. Diagnostic challenges surrounding non-epithelioid MPM are recognised, and there is a critical need for reliable and non-invasive investigative tools in this population.

## Figures and Tables

**Table 1:** Immunohistochemical markers for differentiating tumour types in malignant pleural effusion [12,24]. Adapted from Bibby et al. [44].

Mesothelial Markers	Adenocarcinoma Markers	Other Markers
Calretinin	TTF1 (lung and thyroid)	Squamous cell lung cancer: p40, p63 and claudin 4
CK 5/6	CEA	Renal cell carcinoma: PAX8, PAX2 and claudin 4
WT1	Ber–EP4	Pancreas: CA19-9
D2-40		Gastrointestinal: CD20 and CDX-2
		Gynaecological: PAX-8 and WT1 Prostate: PSA and PSMA
		Breast: mammaglobin, GCDFP-15, ER, PR and GATA3

## References

[1] Woolhouse I., Bishop L., Darlison L., De Fonseka D., Edey A., Edwards J., Faivre-Finn C., Fennell D.A., Holmes S., Kerr K.M. (2018). British Thoracic Society Guideline for the investigation and management of malignant pleural mesothelioma. Thorax.

[2] Sekido Y. (2013). Molecular pathogenesis of malignant mesothelioma. Carcinogenesis.

[3] Wagner J.C., Sleggs C.A., Marchand P. (1960). Diffuse pleural mesothelioma and asbestos exposure in the North Western Cape Province. Br. J. Ind. Med..

[4] Bibby A.C., Tsim S., Kanellakis N., Ball H., Talbot D.C., Blyth K.G., Maskell N.A., Psallidas I. (2016). Malignant pleural mesothelioma: An update on investigation, diagnosis and treatment. Eur. Respir. Rev..

[5] LaDou J., Castleman B., Frank A., Gochfeld M., Greenberg M., Huff J., Joshi T.K., Landrigan P.J., Lemen R., Myers J. (2010). The Case for a Global Ban on Asbestos. Environ. Heal Perspect..

[6] Odgerel C.-O., Takahashi K., Sorahan T., Driscoll T., Fitzmaurice C., Yoko O.M., Sawanyawisuth K., Furuya S., Tanaka F., Horie S. (2017). Estimation of the global burden of mesothelioma deaths from incomplete national mortality data. Occup. Environ. Med..

[7] Testa J.R., Cheung M., Pei J., Below J.E., Tan Y., Sementino E., Cox N.J., Dogan A.U., Pass H.I., Trusa S. (2011). P1 mutations predispose to malignant mesothelioma. Nat. Genet..

[8] Scherpereel A., Opitz I., Berghmans T., Psallidas I., Glatzer M., Rigau D., Astoul P., Bölükbas S., Boyd J., Coolen J. (2020). ERS/ESTS/EACTS/ESTRO guidelines for the management of malignant pleural mesothelioma. Eur. Respir. J..

[9] Robinson B.W.S., Lake R.A. (2005). Advances in Malignant Mesothelioma. N. Engl. J. Med..

[10] Carbone M., Adusumilli P.S., Alexander H.R., Baas P., Bardelli F., Bononi A., Bueno R., Felley-Bosco E., Galateau-Salle F., Jablons D. (2019). Mesothelioma: Scientific clues for prevention, diagnosis, and therapy. CA Cancer J. Clin..

[11] Arnold D.T., Maskell N.A. (2018). Biomarkers in mesothelioma. Ann. Clin. Biochem.

[12] Beckett P., Edwards J., Fennell D., Hubbard R., Woolhouse I., Peake M. (2015). Demographics, management and survival of patients with malignant pleural mesothelioma in the National Lung Cancer Audit in England and Wales. Lung Cancer.

[13] Abo E-Magd G.H., Abouissa A.H., Abbass I. (2019). Diagnostic yield and safety of medical thoraco-scopic versus CT guided percutaneous tru-cut pleural biopsy. Eur. Respir. J..

[14] Roberts M., Neville E., Berrisford R.G., Antunes G., Ali N.J., on behalf of the BTS Pleural Disease Guideline Group (2010). Management of a malignant pleural effusion: British Thoracic Society pleural disease guideline 2010. Thorax.

[15] Kindler H.L., Ismaila N., Armato S.G., Bueno R., Hesdorffer M., Jahan T., Jones C.M., Miettinen M., Pass H., Rimner A. (2018). Treatment of Malignant Pleural Mesothelioma: American Society of Clinical Oncology Clinical Practice Guideline. J. Clin. Oncol.

[16] Physicians RCo (2020). National Mesothelioma Audit Report 2020 (for the Audit Period 2016–18).

[17] Damhuis R., Khakwani A., De Schutter H., Rich A., Burgers J., Van Meerbeeck J. (2015). Treatment patterns and survival analysis in 9014 patients with malignant pleural mesothelioma from Belgium, the Netherlands and England. Lung Cancer.

[18] Bibby A.C., Williams K., Smith S., Bhatt N., A Maskell N. (2016). What is the role of a specialist regional mesothelioma multidisciplinary team meeting? A service evaluation of one tertiary referral centre in the UK. BMJ Open.

[19] Hooper C., Lee Y.C.G., Maskell N. (2010). Investigation of a unilateral pleural effusion in adults: British Thoracic Society pleural disease guideline 2010. Thorax.

[20] Arnold D.T., De Fonseka D., Perry S., Morley A., Harvey J.E., Medford A., Brett M., Maskell N.A. (2018). Investigating unilateral pleural effusions: The role of cytology. Eur. Respir. J..

[21] Porcel J.M. (2018). Biomarkers in the diagnosis of pleural diseases: A 2018 update. Ther. Adv. Respir. Dis..

[22] Hjerpe A., Ascoli V., Bedrossian C.W.M., Boon M.E., Creaney J., Davidson B., Dejmek A., Dobra K., Fassina A., Field A. (2015). Guidelines for the Cytopathologic Diagnosis of Epithelioid and Mixed-Type Malignant Mesothelioma: A secondary publication. Cytopathology.

[23] Monaco S., Mehrad M., Dacic S. (2018). Recent Advances in the Diagnosis of Malignant Mesothelioma: Focus on Approach in Challenging Cases and in Limited Tissue and Cytologic Samples. Adv. Anat. Pathol..

[24] Husain A.N., Colby T.V., Ordóñez N.G., Allen T.C., Attanoos R.L., Beasley M.B., Butnor K.J., Chirieac L.R., Churg A.M., Dacic S. (2018). Guidelines for Pathologic Diagnosis of Malignant Mesothelioma 2017 Update of the Consensus Statement From the International Mesothelioma Interest Group. Arch. Pathol. Lab. Med..

[25] Pinelli V., Laroumagne S., Sakr L., Marchetti G.P., Tassi G.F., Astoul P. (2012). Pleural Fluid Cytological Yield and Visceral Pleural Invasion in Patients with Epithelioid Malignant Pleural Mesothelioma. J. Thorac. Oncol..

[26] Chapel D.B., Schulte J.J., Husain A.N., Krausz T. (2020). Application of immunohistochemistry in diagnosis and management of malignant mesothelioma. Trans. Lung Cancer Res..

[27] Zeren E.H., Demirag F. (2010). Benign and Malignant Mesothelial Proliferation. Surg. Pathol. Clin..

[28] Attanoos R.L., Griffin A., Gibbs A.R. (2003). The use of immunohistochemistry in distinguishing reactive from neoplastic mesothelium. A novel use for desmin and comparative evaluation with epithelial membrane antigen, p53, platelet-derived growth factor-receptor, P-glycoprotein and Bcl-2. Histopathology.

[29] Churg A., Sheffield B.S., Galateau-Salle F. (2016). New Markers for Separating Benign From Malignant Mesothelial Proliferations: Are We There Yet?. Arch. Pathol. Lab. Med..

[30] Ikeda K., Tate G., Suzuki T., Kitamura T., Mitsuya T. (2011). Diagnostic usefulness of EMA, IMP3, and GLUT-1 for the immunocytochemical distinction of malignant cells from reactive mesothelial cells in effusion cytology using cytospin preparations. Diagn. Cytopathol..

[31] Shen J., Pinkus G.S., Deshpande V., Cibas E.S. (2009). Usefulness of EMA, GLUT-1, and XIAP for the cytologic diagnosis of malignant mesothelioma in body cavity fluids. Am. J. Clin. Pathol..

[32] Pulford E., Huilgol K., Moffat D., Henderson D.W., Klebe S. (2017). Malignant Mesothelioma, BAP1 Immunohistochemistry, and VEGFA: Does BAP1 Have Potential for Early Diagnosis and Assessment of Prognosis?. Dis. Markers.

[33] Wang L.-M., Shi Z.-W., Wang J.-L., Lv Z., Du F.-B., Yang Q.-B., Wang Y. (2017). Diagnostic accuracy of BRCA1-associated protein 1 in malignant mesothelioma: A meta-analysis. Oncotarget.

[34] Murali R., Wiesner T., Scolyer R.A. (2013). Tumours associated with BAP1 mutations. Pathology.

[35] Hida T., Matsumoto S., Hamasaki M., Kawahara K., Tsujimura T., Hiroshima K., Kamei T., Taguchi K., Iwasaki A., Oda Y. (2015). Deletion status of p16 in effusion smear preparation correlates with that of underlying malignant pleural mesothelioma tissue. Cancer Sci.

[36] Hida T., Hamasaki M., Matsumoto S., Sato A., Tsujimura T., Kawahara K., Iwasaki A., Okamoto T., Oda Y., Honda H. (2017). Immunohistochemical detection of MTAP and BAP1 protein loss for mesothelioma diagnosis: Comparison with 9p21 FISH and BAP1 immunohistochemistry. Lung Cancer.

[37] Kinoshita Y., Hamasaki M., Yoshimura M., Matsumoto S., Sato A., Tsujimura T., Ueda H., Makihata S., Kato F., Iwasaki A. (2018). A combination of MTAP and BAP1 immunohistochemistry is effective for distinguishing sarcomatoid mesothelioma from fibrous pleuritis. Lung Cancer.

[38] Kinoshita Y., Hida T., Hamasaki M., Matsumoto S., Sato A., Tsujimura T., Kawahara K., Hiroshima K., Oda Y., Nabeshima K. (2018). A combination of MTAP and BAP1 immunohistochemistry in pleural effusion cytology for the diagnosis of mesothelioma. Cancer Cytopathol.

[39] Chapel D.B., Schulte J.J., Berg K., Churg A., Dacic S., Fitzpatrick C., Galateau-Salle F., Hiroshima K., Krausz T., Le Stang N. (2020). MTAP immunohistochemistry is an accurate and reproducible surrogate for CDKN2A fluorescence in situ hybridization in diagnosis of malignant pleural mesothelioma. Mod. Pathol.

[40] Alì G., Bruno R., Poma A.M., Proietti A., Ricci S., Chella A., Melfi F., Ambrogi M.C., Lucchi M., Fontanini G. (2020). A gene-expression-based test can outperform bap1 and p16 analyses in the differential diagnosis of pleural mesothelial proliferations. Oncol. Lett..

[41] Davidson B., Tötsch M., Wohlschlaeger J., Hager T., Pinamonti M. (2018). The diagnostic role of BAP1 in serous effusions. Hum. Pathol..

[42] Selves J., Long-Mira E., Mathieu M.-C., Rochaix P., Ilié M. (2018). Immunohistochemistry for Diagnosis of Metastatic Carcinomas of Unknown Primary Site. Cancers.

[43] Fels E.D.R., Jones K.D. (2020). Diagnosis of Mesothelioma. Surg. Pathol. Clin..

[44] Bibby A.C., Dorn P., Psallidas I., Porcel J.M., Janssen J., Froudarakis M., Subotic D., Astoul P., Licht P., Schmid R. (2019). ERS/EACTS statement on the management of malignant pleural effusions. Eur. J. Cardio-Thoracic Surg..

[45] Ledda C., Senia P., Rapisarda V. (2018). Biomarkers for Early Diagnosis and Prognosis of Malignant Pleural Mesothelioma: The Quest Goes on. Cancers.

[46] Robinson B.W., Creaney J., Lake R., Nowak A., Musk A.W., de Klerk N., Winzell P., Hellstrom K.E., Hellstrom I. (2003). Mesothelin-family proteins and diagnosis of mesothelioma. Lancet.

[47] Creaney J., Robinson B.W.S. (2017). Malignant Mesothelioma Biomarkers: From Discovery to Use in Clinical Practice for Diagnosis, Monitoring, Screening, and Treatment. Chest.

[48] Cui A., Jin X.-G., Zhai K., Tong Z.-H., Shi H.-Z. (2014). Diagnostic values of soluble mesothelin-related peptides for malignant pleural mesothelioma: Updated meta-analysis. BMJ Open.

[49] Pass H.I., Wolaniuk D., Wali A., Thiel R., Hellstrom I., Sardesai N.Y. (2005). Soluble mesothelin related peptides: A potential biomarker for malignant pleural mesothelioma. J. Clin. Oncol..

[50] Davies H.E., Sadler R.S., Bielsa S., Maskell N.A., Rahman N.M., Davies R.J.O., Ferry B.L., Lee Y.C.G. (2009). Clinical Impact and Reliability of Pleural Fluid Mesothelin in Undiagnosed Pleural Effusions. Am. J. Respir. Crit. Care Med..

[51] Hooper C.E., Morley A.J., Virgo P., Harvey J.E., Kahan B., Maskell N.A. (2013). A prospective trial evaluating the role of mesothelin in undiagnosed pleural effusions. Eur. Respir. J..

[52] Hu Z.-D., Liu X.-F., Ding C.-M., Hu C.-J. (2014). Diagnostic accuracy of osteopontin for malignant pleural mesothelioma: A systematic review and meta-analysis. Clin. Chim. Acta.

[53] Pass H.I., Levin S.M., Harbut M.R., Melamed J., Chiriboga L., Donington J., Huflejt M., Carbone M., Chia D., Goodglick L. (2012). Fibulin-3 as a Blood and Effusion Biomarker for Pleural Mesothelioma. New Engl. J. Med..

[54] Creaney J., Dick I.M., Meniawy T., Leong S.L., Leon J.S., Demelker Y., Segal A., Musk A.W.B., Lee Y.C.G., Skates S.J. (2014). Comparison of fibulin-3 and mesothelin as markers in malignant mesothelioma. Thorax.

[55] Kirschner M.B., Pulford E., Hoda M.A., Rozsas A., Griggs K., Cheng Y.Y., Edelman J.J.B., Kao S.C., Hyland R., Dong Y. (2015). Fibulin-3 levels in malignant pleural mesothelioma are associated with prognosis but not diagnosis. Br. J. Cancer.

[56] Ren R., Yin P., Zhang Y., Zhou J., Zhou Y., Xu R., Lin H., Huang C. (2016). Diagnostic value of fibulin-3 for malignant pleural mesothelioma: A systematic review and meta-analysis. Oncotarget.

[57] Pei D., Li Y., Liu X., Yan S., Guo X., Xu X., Guo X. (2017). Diagnostic and prognostic utilities of humoral fibulin-3 in malignant pleural mesothelioma: Evidence from a meta-analysis. Oncotarget.

[58] Tsim S., Kelly C., Alexander L., McCormick C., Thomson F., Woodward R., Foster J.E., Stobo D.B., Paul J., Maskell N.A. (2016). Diagnostic and Prognostic Biomarkers in the Rational Assessment of Mesothelioma (DIAPHRAGM) study: Protocol of a prospective, multicentre, observational study. BMJ Open.

[59] Usuda K., Iwai S., Funasaki A., Sekimura A., Motono N., Matoba M., Doai M., Yamada S., Ueda Y., Uramoto H. (2019). Diffusion-Weighted Imaging Can Differentiate between Malignant and Benign Pleural Diseases. Cancers.

[60] Leung A.N., Müller N.L., Miller R.R. (1990). CT in differential diagnosis of diffuse pleural disease. Am. J. Roentgenol..

[61] Blyth K.G., Murphy D. (2018). Progress and challenges in Mesothelioma: From bench to bedside. Respir. Med..

[62] Treglia G., Sadeghi R., Annunziata S., Lococo F., Cafarotti S., Bertagna F., Prior J.O., Ceriani L., Giovanella L. (2014). Diagnostic accuracy of 18F-FDG-PET and PET/CT in the differential diagnosis between malignant and benign pleural lesions: A systematic review and meta-analysis. Acad. Radiol..

[63] DeFonseka D. PET-CT in the Undiagnosed Effusion: Results of the TARGET Study. Proceedings of the British Thoracic Society Winter Meeting.

[64] Tsim S., Cowell G.W., Kidd A., Woodward R., Alexander L., Kelly C., E Foster J., Blyth K.G. (2020). A comparison between MRI and CT in the assessment of primary tumour volume in mesothelioma. Lung Cancer.

[65] Lacerenza S., Ciregia F., Giusti L., Bonotti A., Greco V., Giannaccini G., D’Antongiovanni V., Fallahi P., Pieroni L., Cristaudo A. (2020). Putative Biomarkers for Malignant Pleural Mesothelioma Suggested by Proteomic Analysis of Cell Secretome. Cancer Genom. Proteom..

[66] Sage A.P., Martinez V.D., Minatel B.C., Pewarchuk M.E., Marshall E.A., Macaulay G.M., Hubaux R., Pearson D.D., Goodarzi A.A., Dellaire G. (2018). Genomics and Epigenetics of Malignant Mesothelioma. High Throughput.

[67] Joseph N.M., Chen Y.-Y., Nasr A., Yeh I., Talevich E., Onodera C., Bastian B., Rabban J.T., Garg K., Zaloudek C. (2017). Genomic profiling of malignant peritoneal mesothelioma reveals recurrent alterations in epigenetic regulatory genes BAP1, SETD2, and DDX3X. Mod. Pathol..

[68] Cakiroglu E., Senturk S. (2020). Genomics and Functional Genomics of Malignant Pleural Mesothelioma. Int. J. Mol. Sci..

[69] Weber D.G., Gawrych K., Casjens S., Brik A., Lehnert M., Taeger D., Pesch B., Kollmeier J., Bauer T.T., Johnen G. (2017). Circulating miR-132-3p as a Candidate Diagnostic Biomarker for Malignant Mesothelioma. Dis. Markers.

[70] Zhou X.-M., He C.-C., Liu Y.-M., Zhao Y., Zhao D., Du Y., Zheng W.-Y., Li J.-X. (2012). Metabonomic classification and detection of small molecule biomarkers of malignant pleural effusions. Anal. Bioanal. Chem..

[71] Zennaro L., Vanzani P., Nicolè L., Cappellesso R., Fassina A. (2017). Metabonomics by proton nuclear magnetic resonance in human pleural effusions: A route to discriminate between benign and malignant pleural effusions and to target small molecules as potential cancer biomarkers. Cancer Cytopathol..

[72] Tsim S., Paterson S., Cartwright D., Fong C.J., Alexander L., Kelly C., Holme J., Evison M., Blyth K.G. (2019). Baseline predictors of negative and incomplete pleural cytology in patients with suspected pleural malignancy—Data supporting ‘Direct to LAT’ in selected groups. Lung Cancer.

